# A Notch signaling-related lncRNA signature for predicting prognosis and therapeutic response in clear cell renal cell carcinoma

**DOI:** 10.1038/s41598-023-48596-2

**Published:** 2023-11-30

**Authors:** Lulu Zhang, Yulei Li, Bin Cai, Jiajun Chen, Keyuan Zhao, Mengyao Li, Juan Lang, Kaifang Wang, Shouhua Pan, Ke Zhu

**Affiliations:** 1 Department of Medical Research Center, Shaoxing People’s Hospital, No.568, Zhongxing North Road, Shaoxing, 312000 Zhejiang Province China; 2 Department of Urology, Shaoxing People’s Hospital, No.568, Zhongxing North Road, Shaoxing, 312000 Zhejiang Province China; 3 Shaoxing People’s Hospital, No.568, Zhongxing North Road, Shaoxing, 312000 Zhejiang Province China; 4 Department of Pathology, Shaoxing People’s Hospital, No.568, Zhongxing North Road, Shaoxing, 312000 Zhejiang Province China; 5 Faculty of Health Sciences, University of Macau, Taipa, Macau; 6 Nanchang People’s Hospital, No.1268 Jiuzhou Street, Xihu District, Nanchang City, China

**Keywords:** Renal cancer, Drug development, Tumour biomarkers

## Abstract

Increasing evidence has confirmed the vital role of Notch signaling in the tumorigenesis of clear cell renal cell carcinoma (ccRCC). The underlying function of long non-coding RNA (lncRNA) related to Notch signaling in ccRCC remains unclear. In present study, the prognostic value and therapeutic strategy of Notch signaling-related lncRNA are comprehensively explored in ccRCC. In total, we acquired 1422 NSRlncRNAs, of which 41 lncRNAs were identified the key NSRlncRNAs associated with the occurrence of ccRCC. The prognostic signature containing five NSRlncRNAs (AC092611.2, NNT-AS1, AGAP2-AS1, AC147651.3, and AC007406.3) was established and validated, and the ccRCC patients were clustered into the high- and low-risk groups. The overall survival of patients in the low-risk group were much more favorable than those in the high-risk group. Multivariate Cox regression analysis indicated that the risk score was an independent prognostic biomarker. Based on the risk score and clinical variables, a nomogram for predicting prognosis of ccRCC patients was constructed, and the calibration curves and DCA curves showed the superior predictive ability of nomogram. The risk score was correlated with immune cell infiltration, targeted therapy or chemotherapy sensitivity, and multiple oncogenic pathways. Additionally, consensus clustering analysis stratified the ccRCC patients into four clusters with obvious different outcomes, immune microenvironments, and expression of immune checkpoints. The constructed NSRlncRNA-based signature might serve as a potential biomarker for predicting prognosis and response to immunotherapy or targeted therapy in patients with ccRCC.

## Introduction

Renal cancer was a common malignant genitourinary system tumor that ranked the sixth in incidence among men and the ninth in incidence among women in the United States in 2021, accounting for approximately 4% for incidence in all adult cancers^1^. The predominant pathological subtype of renal cancer was clear cell renal cell carcinoma (ccRCC), which was characterized by the high distant metastases and mortality compared to other types of renal cancer, comprising of the main deaths of renal cancer^2^. Therefore, identifying reliable biomarkers for predicting the outcomes and facilitating clinical strategies were of significant clinical meaning.

A highly evolutionarily conserved signaling pathway, Notch, first identified by John Dexter and Thomas Morgan in Drosophila^3^, has been received considerable attention owing to the significant roles in regulating embryonic development^4,5^ cell growth and death^6,7^, homeostasis^8,9^, inflammation^10^, angiogenesis^11^, autophagy^12^, and metabolism^13^. The key components of the Notch signaling were four receptors (Notch1, Notch2, Notch3, and Notch4), five ligands (JAG1, JAG2, DLL1, DLL3, and DLL4), and several target genes. Receptor-ligand binding stimulated the activation of Notch signaling, followed with the hydrolyzation of Notch receptors by ADAM and γ-secretase to generate Notch intracellular domain (NICD), which further involved in the transcriptional regulation of downstream genes, such as HES1, HEYL, and KAT2A. Accumulating studies have confirmed that carcinogenesis, cancer progression, and drug resistance were generally accompanied by the aberrant regulation of the Notch signaling pathway. Silencing NOTCH1 attenuated the proliferation and invasion of ccRCC cells^14^. The blockade of Notch signaling could effectively inhibit RCC growth^15^. The expressions of Notch1, Notch2, JAG1, JAG2, DLL1, and DLL4 were markedly elevated in RCC stem cells. Moreover, Notch signaling facilitated the tumor stem cells resistance to cisplatin and sorafenib via activating the SDF-1/CXCR4 axis^15^.

Long non-coding RNAs (lncRNAs) are a type of transcript RNAs consisting of more than 200 nucleotides. LncRNA was initially treated as the transcriptional noise and has no biological function. Up to now, increasing evidence have demonstrated that lncRNA was implicated in various physiological processes and pathological events through epigenetic, transcriptional and posttranscriptional regulation, such as metabolism^16–18^, autophagy^19–21^, inflammation^22^, and immune^23–25^. LncRNA CEBPA-AS1 overexpression attenuated the malignant progression of osteosarcoma by reducing the activity of Notch signaling pathway LncRNA BASP1‐AS1 advanced the melanoma progression by interacting with YBX1 to activate Notch signaling^26^ LncRNA UCA1 accelerated the malignant phenotypes of ccRCC cells via sponging miR-182-5p to positively regulate the expression of DLL4^27^. However, the potential function and prognostic values of Notch signaling-related lncRNA in ccRCC was still unclear.

In the current study, the underlying correlations of Notch signaling-related lncRNAs with outcomes and therapeutic response in patients with ccRCC were investigated. With the integrated application of TCGA database and the Notch signaling, a prognostic signature consisting of five Notch signaling-related lncRNAs (AC092611.2, NNT-AS1, AGAP2-AS1, AC147651.3, and AC007406.3) was established. Furthermore, the correlation between the prognostic signature and clinicopathological variables was explored, and several variables associated with outcomes were performed to establish a nomogram for predicting the prognosis. Additionally, the relationship between the prognostic signature and tumor microenvironment, immune checkpoints, and sensitivity to targeted drugs or chemotherapeutic drugs were also explored. Overall, we aimed to identify novel biomarkers for the valid prediction of outcomes and therapeutic efficacy in ccRCC patients.

## Methods

### Acquisition of the Notch signaling-related lncRNA

**Figure 1:**
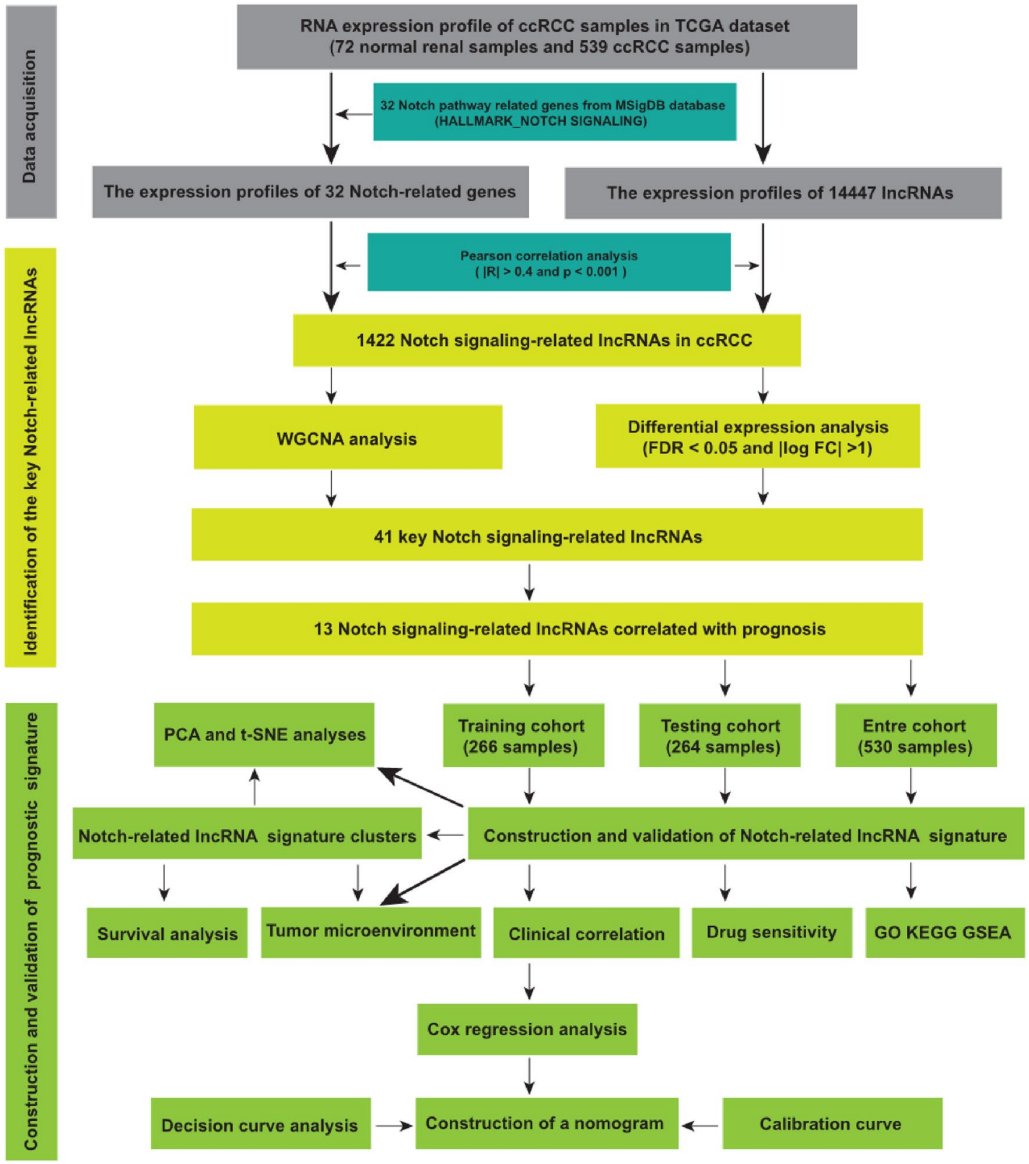
The flow chart of the present study.

We downloaded the transcriptional expression profile as fragments per kilobase of transcript per million mapped reads (FPKM), clinicopathological characteristics, and survival-related data for patients with clear cell renal cell cancer (ccRCC) from the Cancer Genome Atlas database (TCGA, https://portal.gdc.cancer.gov/). Totally, we have acquired the RNA sequencing data of 611 samples (539 ccRCC samples and 72 normal renal samples) and the corresponding clinicopathological variables including age, AJCC stage, gender, and grade. The lncRNA annotation data of GRCh38 was provided by the GENCODE database (https://www.gencodegenes.org). The 32 Notch pathway-related genes were obtained from the “HALLMARK_NOTCH_SIGNALING” gene set of the Molecular Signatures Database version 7.5 (MSigDB, https://www.gsea-msigdb.org/GSEA/misgdb). In addition, the correlation between Notch signaling genes and Notch signaling-related lncRNAs (NSRlncRNAs) was weighed by Pearson correlation analysis, and the NSRlncRNAs were identified with the criterion that the correlation coefficient (| R2 |> 0.4) and *p* < 0.001. The overall workflow of the current study was depicted in Fig. 1.

### Identification of the key Notch signaling-related lncRNAs

Weighted gene co-expression network analysis (WGCNA), as a crucial bioinformatics approach, was conducted to investigate the NSRlncRNAs between various samples and identify lncRNA modules notably correlated with carcinogenesis by using R package “WGCNA”. To screen the most cancer-associated modules, we performed the module-trait correlation analyses for each module. Subsequently, the NSRlncRNAs from the crucial cancer-correlated modules were used for further analysis. In addition, the differentially expressed NSRlncRNAs (DENSRlncRNAs) between ccRCC tissues and normal renal tissues was identified through the application of R package “edgeR” with the criterion of false discovery rate (FDR) < 0.05 and ∣log fold change (FC) |> 1. Finally, Draw Venn Diagram (http://bioinformatics.psb.ugent.be/webtools/Venn/), as an online website, was conducted to acquire the overlapping lncRNAs and identify the key NSRlncRNAs.

### Construction and verification of Notch signaling-related lncRNA signature

Patients with ccRCC in the total dataset (n = 530) were randomly classified into the training dataset (n = 266) or testing dataset (n = 264) with a ratio 1:1. The training dataset was applied to establish the prognostic signature, while the testing dataset and total dataset were used to validate the constructed signature. The univariate Cox regression analysis was applied to identify NSRlncRNA correlated with prognosis (*p* < 0.05), which were subsequently incorporated for the least absolute shrinkage and selection operator (LASSO) regression analysis, followed by the establishment of the Notch-related lncRNA-based risk signature. The risk score of each ccRCC patient was generated via the following formula: risk score = (βNSRlncRNA1 × expNSRlncRNA1) + (βNSRlncRNA2 × expNSRlncRNA2) + ••• + (βNSRlncRNAn × expNSRlncRNAn). The βNSRlncRNA referred to the relevant coefficient of NSRlncRNA by LASSO analysis, and expNSRlncRNA referred to the expression of NSRlncRNA. Based on the median risk score, ccRCC patients would be classified into high- and low-risk groups. Principal component analysis (PCA) and t-distributed stochastic neighbor embedding (t-SNE) were performed for the analysis of reducing dimension in exploring the power of clustering ability about the prognostic signature. K-M curves and time-dependent receiver operating characteristic (ROC) curves were conducted to evaluate the predictive power of our constructed signature in prognosis.

### Construction of the nomogram

First, we explored the correlation between the prognostic signature and clinical variables (age, gender, AJCC stage, and grade). Second, we performed univariate and multivariate Cox regression analyses for investigating whether the prognostic signature was of independent prognostic value in patients with ccRCC. Finally, based on the result of multivariate COX regression analysis, a nomogram consisting of several clinicopathological variables and risk score was established. We drew the nomogram to forecast the 1-, 3-, and 5-year survival probabilities of ccRCC patients by using R package “rms”. In addition, the calibration curve and decision curve analysis (DCA) were plotted to estimate the predictive performance of the nomogram.

### Tumor immune microenvironment analysis

ESTIMATE algorithm was conducted to investigate the relationship between the NSRlncRNA-based signature and immune microenvironment. In addition, the CIBERSORT, CIBERSORT-ABS, EPIC, MCP counter, TIMER, and XCELL algorithms were performed to assess the relative abundance of 22 immune cell types among the low- and high-risk groups. Moreover, we also analyzed the expression levels of some key immune checkpoints among two groups.

### Functional enrichment analysis

Gene set enrichment analysis (GSEA) was conducted to uncover the underlying functional pathways of the Notch-correlated lncRNA prognostic signature via performing GSEA software (Version 4.1.0), and FDR < 0.25 represented significantly enriched pathways. Additionally, the differentially expressed genes (DEGs) were identified among two risk groups with the criterion of FDR < 0.05 and ∣log FC |> 1. Subsequently, we performed the Gene Ontology (GO) and Kyoto Encyclopedia of Genes and Genomes^28–30^ (KEGG) analyses to explore the potential cancer biology based on the results of the differentially expressed analysis with the criterion of *p* < 0.05.

### Identification of candidate small molecule drugs

The differentially expressed genes based on the NSRlncRNA-based signature were classified into upregulated gene group and downregulated gene group. Subsequently, these genes were separately submitted into the L1000FWD website (https://maayanlab.cloud/L1000FWD/), and the corresponding results were acquired.

### Drug sensitivity prediction

The Genomics of Drug Sensitivity in Cancer (GDSC, http://www.cancerrxgene.org) database was conducted to predict the response to common drugs of ccRCC patients in two groups. The sensitivity was assessed via calculating the half-maximal inhibitory concentration (IC50) using R package “pRRophetic”. *P*-value < 0.05 was deemed as statistical criteria..

### Consensus Clustering

In the total TCGA cohort, based on the expression levels of risk score, hierarchical clustering was conducted by using the R package “ConsensusClusterPlus”. PCA and t-SNE analyses were conducted to further confirm the clustering power. Furthermore, we also explored the difference of survival outcomes, immune microenvironment, expression of immune checkpoint, and immune cell infiltration among the clusters.

### Statistical analysis

All statistical analyses were conducted based on R software (Version 4.0.5). *P* < 0.05 was served as statistical criteria.

## Results

### Identification of the key Notch signaling-related lncRNA

**Figure 2:**
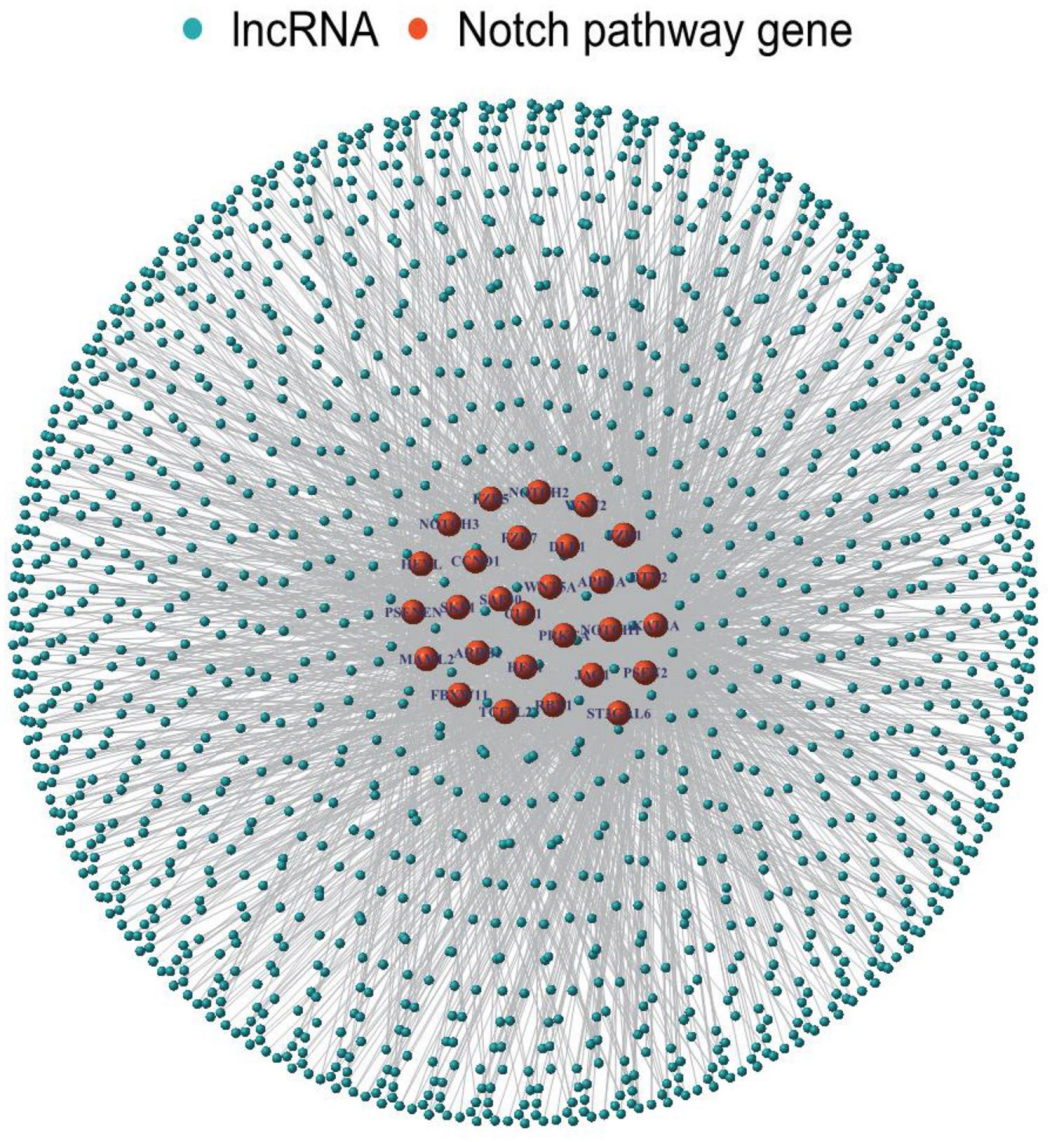
The network of the correlations between Notch signaling-related genes and lncRNA.

**Figure 3:**
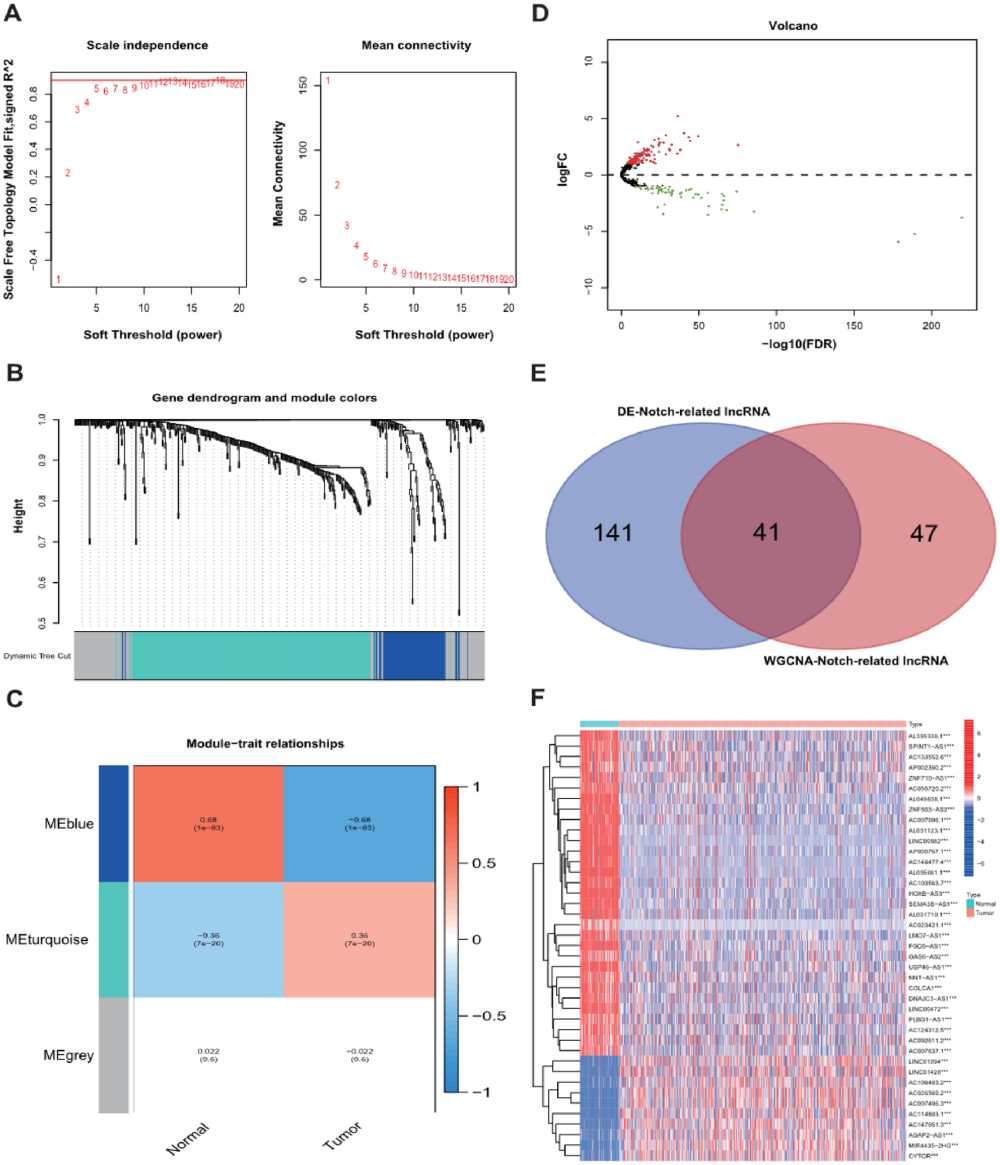
Identification of the key Notch-related lncRNA. (**A**) The network topographies with various soft threshold powers; (**B**) Dendrogram of Notch-related lncRNA clustered on the basis of a dissimilarity measure; (**C**) The correlation between the clinical traits and Notch-related lncRNA; (**D**) Volcano map for the differentially expressed Notch- related lncRNA; (**E**) Venn diagram for identification of the key Notch-related lncRNA; (**F**) Heatmap showed the key Notch-related lncRNA between normal samples and tumor samples.

In the TCGA dataset, a total of 1422 lncRNAs showed the significant correlation with 32 Notch signaling genes and were defined as NSRlncRNA. The relevant co-expression network between lncRNA and Notch signaling genes was shown in Fig. 2. Subsequently, in order to discovery the vital module of NSRlncRNA in ccRCC, WGCNA analysis was conducted in the TCGA dataset, and the optimal threshold value was 10 if the correlational coefficient was > 0.9 (Fig. 3A). These NSRlncRNAs were classified by the average linkage hierarchical clustering approach, and three modules were generated (Fig. 3B). Furthermore, the blue module containing 88 NSRlncRNAs was negatively correlated with ccRCC tissues (r = − 0.68, *p* = 1e-83) and used for further analysis (Fig. 3C). Meanwhile, 182 DENSRlncRNAs containing 119 upregulated NSRlncRNAs and 63 downregulated NSRlncRNAs, were identified between ccRCC samples and normal samples (Fig. 3D). Finally, The Venn diagram was performed to obtain the 41 key NSRlncRNAs (Fig. 3E), namely the intersection of the result of WGCNA analysis and DENSRlncRNAs, which were showed by the heatmap (Fig. 3F).

### Establishment of the prognostic signature

**Figure 4:**
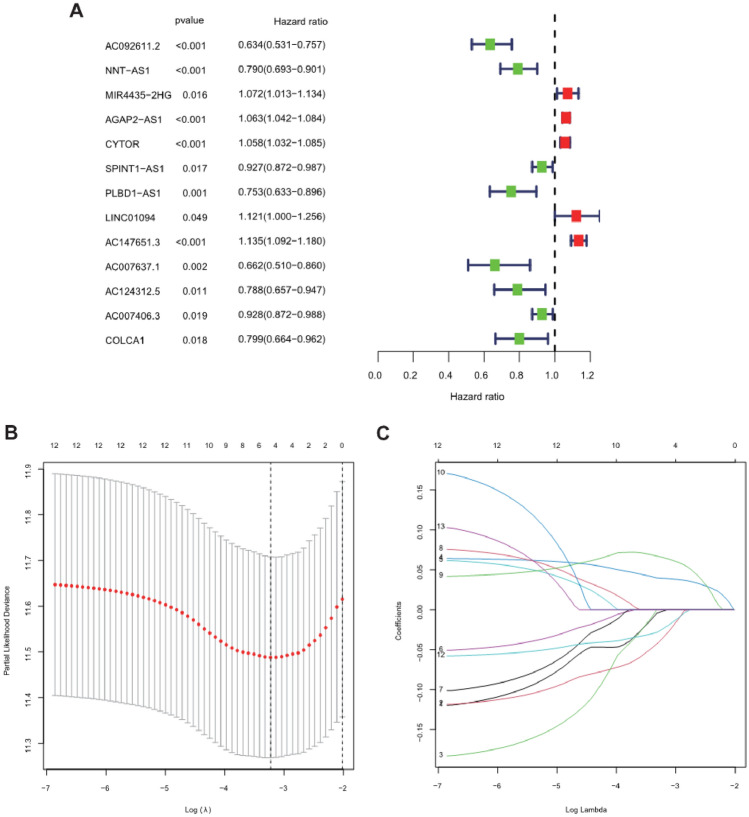
Identification of the prognostic Notch-related lncRNA. (**A**) Identification of the prognostic lncRNA by univariate Cox regression analysis (*P* < 0.05); (**B**) Tenfold cross-validation for tuning in the LASSO analysis; (**C**) The coefficient profile of five by LASSO regression analysis.

**Figure 5:**
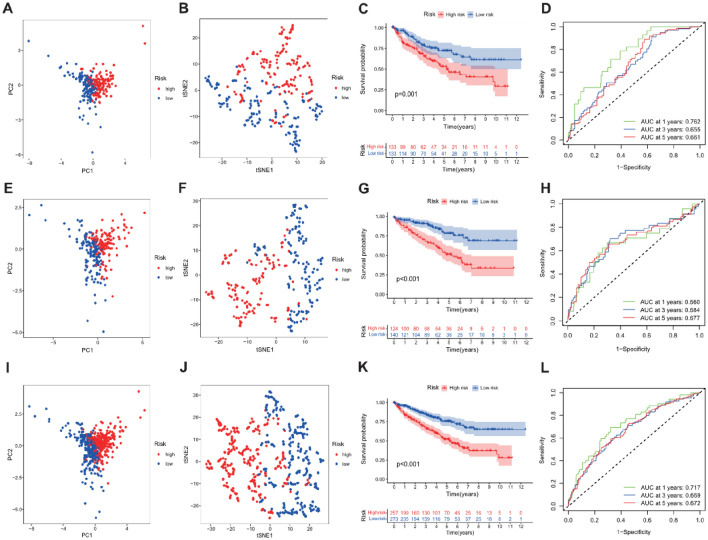
Construction and validation of the Notch-related lncRNA signature. PCA analysis in the training TCGA dataset (**A**), testing TCGA dataset (**E**), and total TCGA dataset (**I**); t-SNE analysis in the training TCGA dataset (**B**), testing TCGA dataset (**F**), and total TCGA dataset (**J**); Kaplan–Meier curve indicated that the low-risk patients had better prognosis compared with the high-risk patients in the training TCGA dataset (**C**), testing TCGA dataset (**G**), and total TCGA dataset (**K**); Time-independent receiver operating characteristic (ROC) analysis for evaluating the predictive ability of Notch-related lncRNA signature in the training TCGA dataset (**D**), testing TCGA dataset (**H**), and total TCGA dataset (**L**).

**Figure 6:**
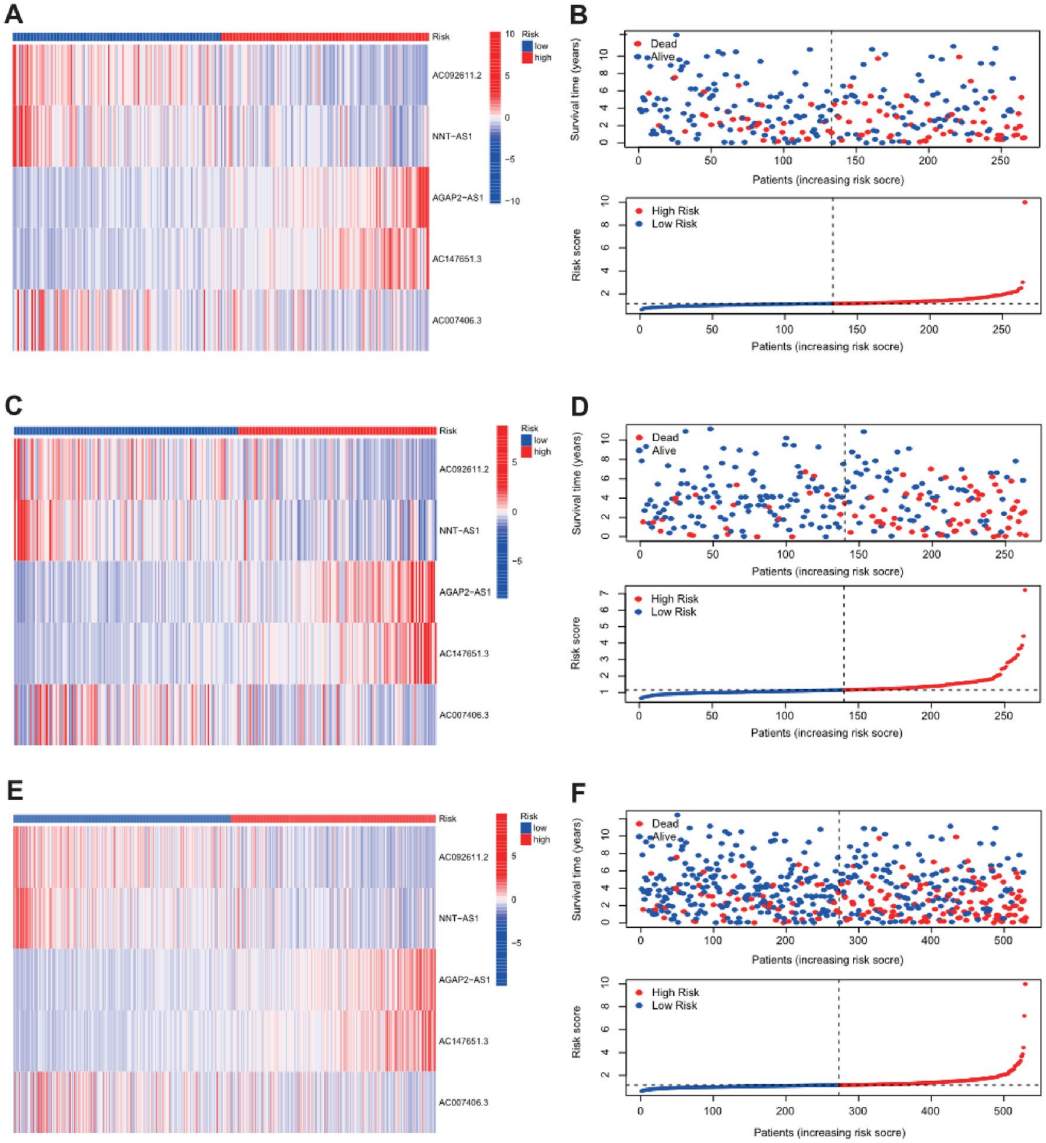
Heatmap showed the expression of prognostic Notch-related lncRNA between the high-risk group and low-risk group in the training TCGA dataset (**A**), testing TCGA dataset (**C**), and total TCGA dataset (**E**); Distribution of risk scores and survival status for the ccRCC patients based on the Notch-related lncRNA signature in the training TCGA dataset (**B**), testing TCGA dataset (**D**), and total TCGA dataset (**F**).

Univariate Cox regression analysis was applied to screen the prognostic lncRNA from 41 key NSRlncRNAs in the training cohort, and 13 prognostic NSRlncRNAs were identified (*p* < 0.05) (Fig. 4A). To avert the risk of over-fitting, these NSRlncRNAs were incorporated into LASSO regression analysis for developing the predictive signature (Fig. 4B,C), of which five NSRlncRNAs were utilized to establish the signature. The risk score = (-0.002 × AC092611.2 expression) + (− 0.0359 × NNT-AS1 expression) + (0.0395 × AGAP2-AS1 expression) + (0.0656 × AC147651.3 expression) + (− 0.0243 × AC007406.3 expression). Based on the median risk score generated via the previous formula, 266 patients in the training cohort were clustered into two different groups (high-risk group and low-risk group). PCA and t-SNE analyses displayed the excellent clustering power of NSRlncRNA-based risk score (Fig. 5A,B). Kaplan–Meier curves showed the much longer overall survival of ccRCC patients in the low-risk group compared with the high-risk group (*p* = 0.001; Fig. 5C), with the 1-, 3-, and 5-year AUC values of 0.762, 0.655, and 0.661, respectively (Fig. 5D). The distribution and survival status of ccRCC patients stratified by the median risk score were shown in the Fig. 6B. The expression patterns of 5 NSRlncRNAs among the low-risk group and the high-risk group were displayed in Fig. 6A. Furthermore, we also discovered the similar results in the testing cohort (Figs. 5E–H, and 6C,D) and the total cohort (Figs. 5I–L, and 6E,F).

### Construction of a nomogram

**Figure 7:**
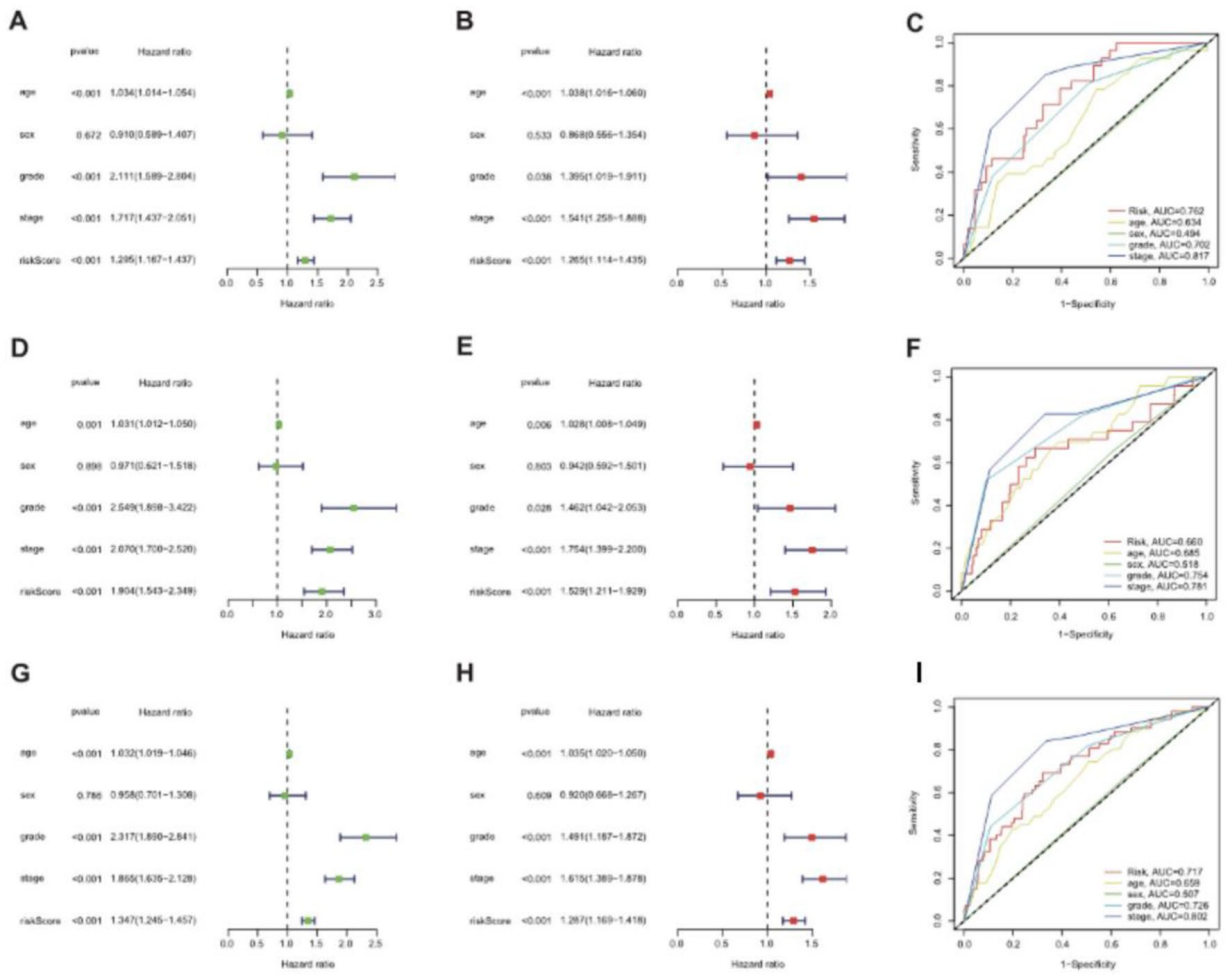
The Notch-related lncRNA signature was an independent prognostic index for patients with ccRCC. The univariate Cox analysis for evaluating the prognostic value of Notch-related lncRNA signature in the training TCGA dataset (**A**), testing TCGA dataset (**D**), and total TCGA dataset (**G**); (**B**) Multivariate Cox analysis for evaluating the independent prognostic value of Notch-related lncRNA signature in the training TCGA dataset (**B**), testing TCGA dataset (**E**), and total TCGA dataset (**H**); (**C**) ROC curves analyses of the clinical variables and risk score in the training TCGA dataset (**C**), testing TCGA dataset (**F**), and total TCGA dataset (**I**).

**Figure 8:**
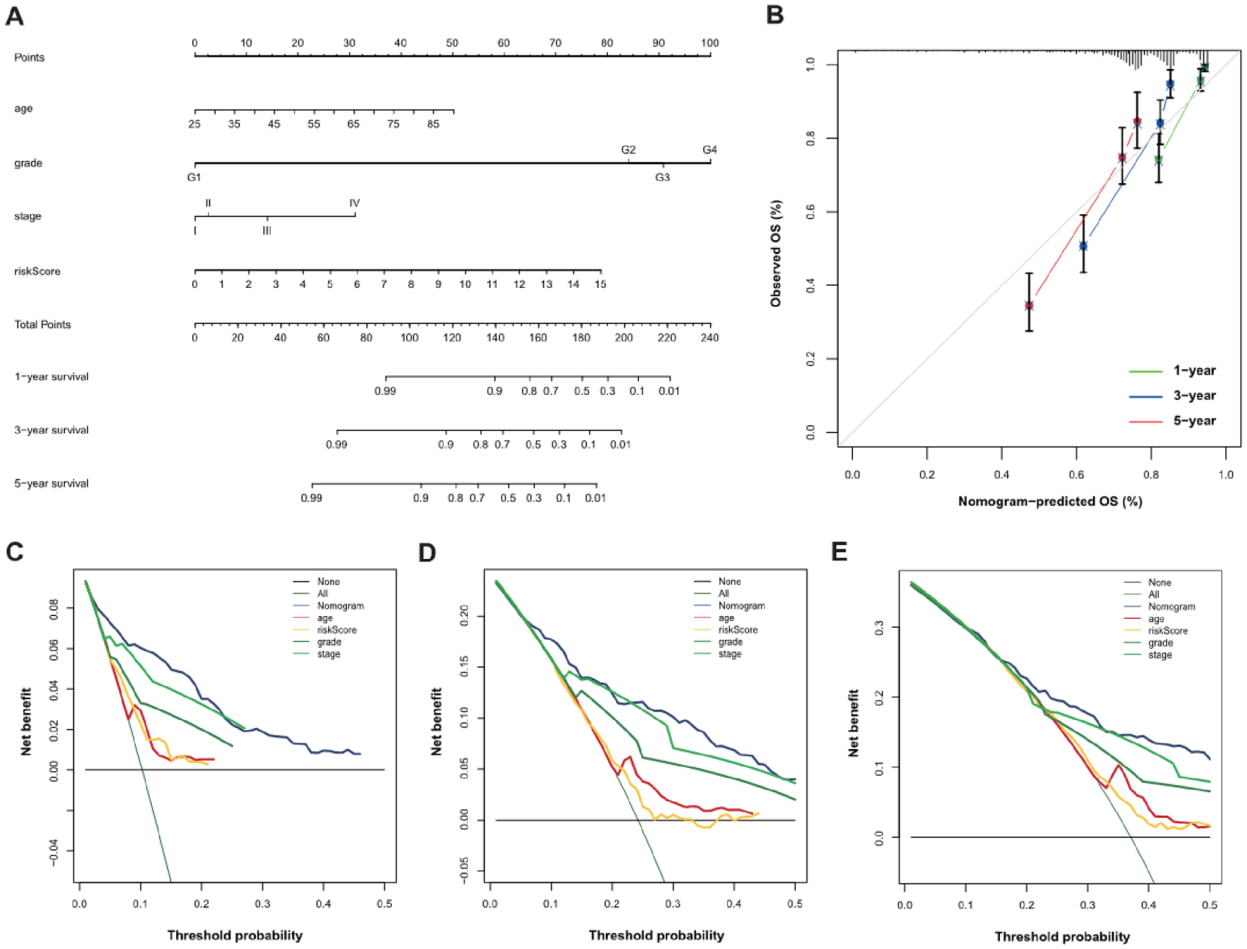
Establishment of the nomogram in the total TCGA dataset. (**A**) Nomogram based on the risk score and clinical variables for predicting 1-year, 3-year, and 5-year survival between ccRCC patients; (**B**) The calibration curves of the nomogram showed the concordance between predicted and actual 1-, 3-, and 5-year survival; (**C**-**E**) Decision curve analysis for evaluating the clinical utility of the nomogram in 1-year, 3-year, and 5-year, respectively.

To validate the clinical value of the signature, we explored whether risk score could be served as an independent prognostic index in ccRCC. The clinical variables (age, sex, grade, and AJCC stage) and risk score were incorporated for the univariate and multivariate COX regression analysis, and the results indicated that risk score was an independent prognostic index for patients with ccRCC in the training cohort, testing cohort, and total cohort (Fig. 7). To facilitate the clinical application of the risk score in prognosis, a nomogram was established (Fig. 8A). Additionally, both calibration curves and DCA curves showed the favorable performance of nomogram in predicting prognosis for patients with ccRCC (Fig. 8B–E). Moreover, we also explored the correlation between risk score and clinical variables, and discovered that the risk score was closely related to advanced clinical variables and significantly elevated in subgroups of grade (G3 and G4), male, and AJCC stage (Stage III and Stage IV) (Supplementary Fig. 1).

### Immune cell infiltration

**Figure 9:**
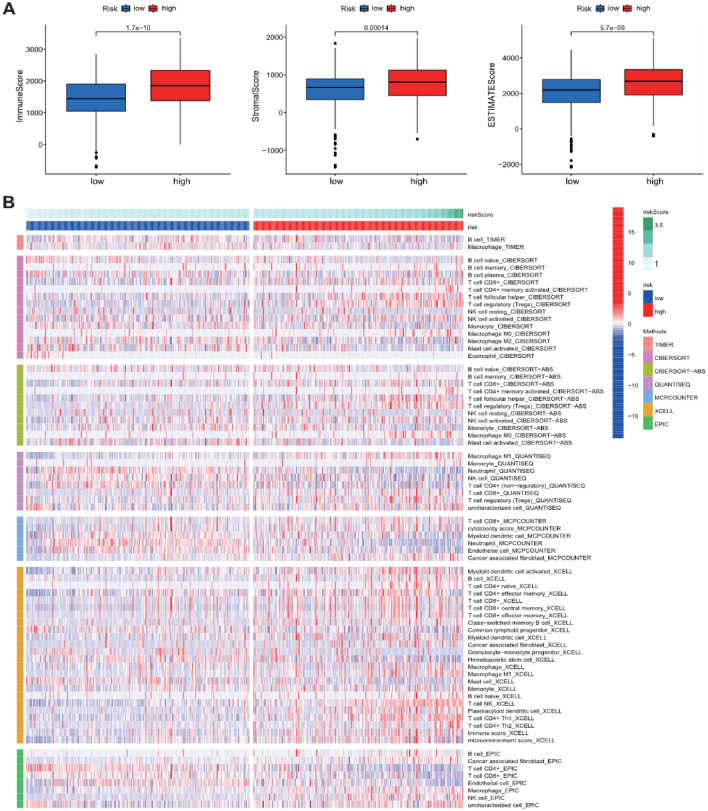
The relationship between the Notch-related lncRNA signature and tumor microenvironment. (**A**) Boxplot showed the expression of ESTIMATE Score, Immune Score, Stromal Score among high-risk group and low-risk group via the ESTIMATE algorithm; (**B**) Heatmap showed the immune cells infiltration among high-risk group and low-risk group by CIBERSORT, QUANTISEQ, MCPcounter, XCELL, CIBERSORT-ABS, TIMER and EPIC algorithms.

Given the significance of immune microenvironment and immune checkpoint inhibitor therapy in ccRCC, we explored the relationship between immune microenvironment and the prognostic signature by CIBERSORT, CIBERSORT-ABS, ESTIMATE, EPIC, MCP counter, TIMER, and XCELL algorithms, and the relevant results were shown in Fig. 9B. The results of ESTIMATE analysis suggested the obvious difference of the immune score, stromal score, and ESTIMATE score among the high- and low-risk groups (Fig. 9A). Moreover, the expression of several immune checkpoints, such as PDCD1, LAG3, CD274, HAVCR2, LAG3, and TIGIT was distinctly different among two groups (Supplementary Fig. 2). All these results suggested that NSRlncRNA-based signature might be implicated in the ccRCC progression via regulating the immune cell infiltration and offering a novel treatment strategy for patients with ccRCC.

### Functional enrichment analysis

Considering the correlation between the NSRlncRNA-based signature and prognosis as well as clinical variables, we further explored the biological functional mechanisms among high- and low-risk groups. GSEA suggested that NSRlncRNA-based signature was notably associated with mTOR signaling pathway, ERBB signaling pathway, the regulation of autophagy, insulin signaling pathway, and peroxisome, fatty acid metabolism, propanoate metabolism, and sphingolipid metabolism (Supplementary Fig. 3A). The results of KEGG^28–30^ analysis indicated that DEGs among two groups were mainly associated with cytokine-cytokine receptor interaction, ECM − receptor interaction, PI3K-Akt and IL-17 signaling pathways, complement and coagulation cascades, and NF − kB signaling pathway (Supplementary Fig. 3C). The results of GO analysis indicated that DEGs among two groups were mainly implicated in the extracellular matrix organization, extracellular structure organization, collagen metabolic process, chemokine activity, basement membrane, peptidase regulator activity, and so on (Supplementary Fig. 3B).

### Identification of the candidate drug

To further identify the candidate therapeutic drugs for ccRCC, we submitted the upregulated genes and downregulated genes among high- and low-risk groups into the L1000FWD database. The top ten significant candidate compounds were served as the therapeutic drugs for the treatment of ccRCC and might enhance the anticancer effect. (Table 1). In addition, we found that these drugs were mainly enriched in the dopamine receptor antagonist, FLT3 inhibitor/JAK inhibitor, EGFR inhibitor, PI3K inhibitor, glycogen synthase kinase inhibitor, and NF-kB pathway inhibitor, which might provide novel directions for developing targeted drugs.

**Table 1:** Identification of the top 10 candidate drugs by using L1000FWD database.

Drug	Similarity score	*p*-value	q-value	Z-score	Combined score	MOA	Predicted MOA
BRD-K92301463	− 0.1111	2.01E-14	2.02E-10	1.81	− 24.81	Unknow	Dopamine receptor antagonist
TG-101348	− 0.1111	6.31E-13	1.92E-09	1.73	− 21.16	FLT3 inhibitor/JAK inhibitor	
Cycloheximide	− 0.0992	1.59E-12	2.62E-09	1.89	− 22.31	Protein synthesis inhibitor	
K784-3131	− 0.0992	5.65E-12	7.33E-09	1.76	− 19.84	Unknow	EGFR inhibitor
TWS-119	− 0.0992	1.77E-11	1.57E-08	1.6	− 17.23	Glycogen synthase kinase inhibitor	
BRD-K32644160	− 0.0992	1.77E-11	1.57E-08	1.71	− 18.39	Unknow	P13K inhibitor
H-7	− 0.0992	2.01E-11	1.69E-08	1.68	− 18	Unknow	Dopamine receptor antagonist
BRD-K74163137	− 0.0913	2.31E-11	1.88E-08	1.75	− 18.6	Unknow	Dopamine receptor antagonist
BRD-K00313977	− 0.1032	2.82E-11	2.11E-08	1.71	− 18.05	Unknow	Dopamine receptor antagonist
BRD-A62200266	− 0.0952	3.12E-11	2.16E-08	1.75	− 18.41	Unknow	NFkB pathway inhibitor

### Drug sensitivity prediction

**Figure 10:**
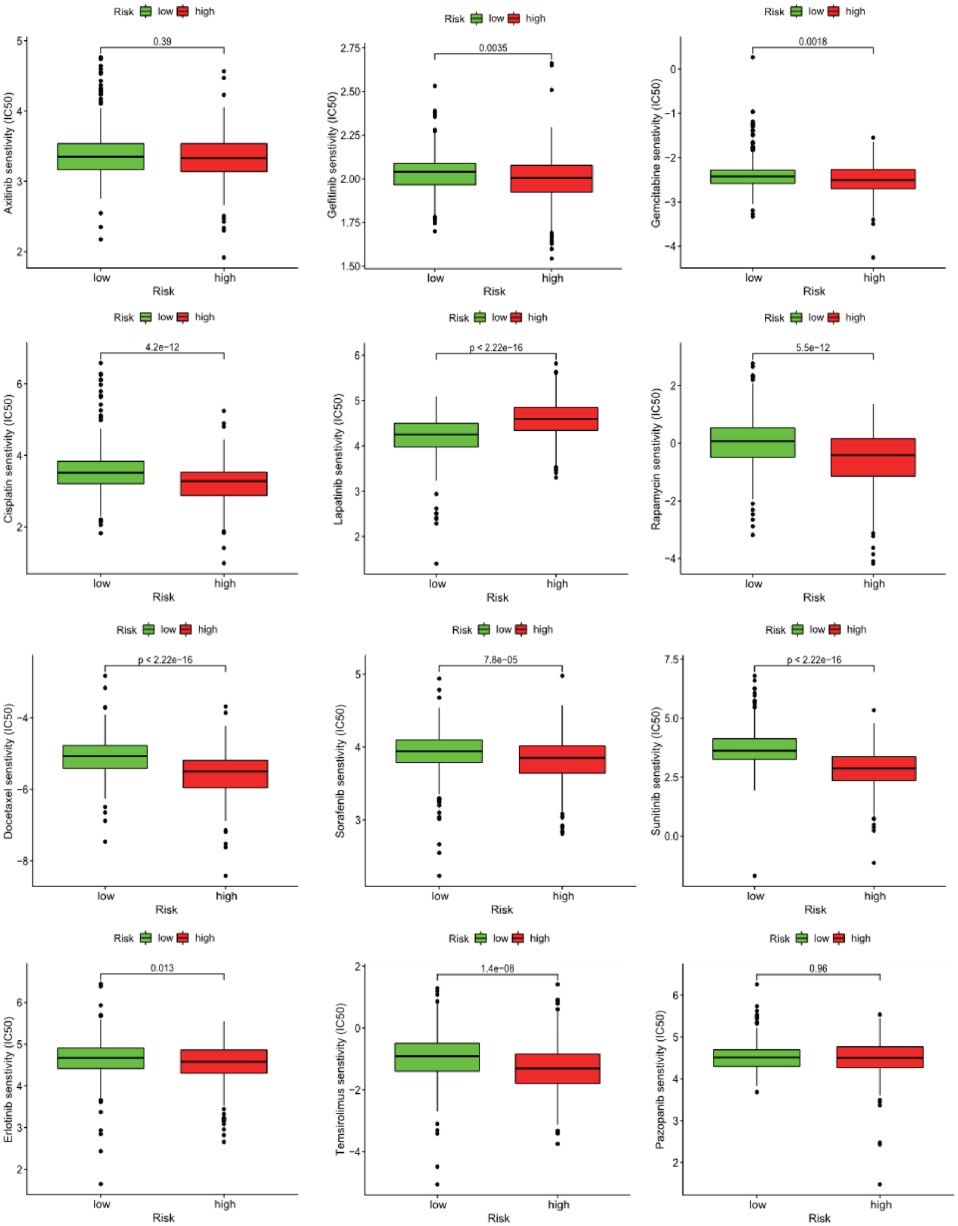
Comparing the estimated half-maximal inhibitory concentration (IC50) of Gefitinib, Sorafenib, Sunitinib, Temsirolimus, Erlotinib, Cisplatin, Gemcitabine, Docetaxel, Rapamycin, Lapatinib, Axitinib, and Pazopanib between the high- and low-risk groups in the total TCGA dataset.

Figure 10 uncovered the results of some commonly targeted or chemotherapy drugs for ccRCC. Our results revealed that the IC50 levels of Gefitinib, Sorafenib, Sunitinib, Temsirolimus, Erlotinib, Cisplatin, Gemcitabine, Docetaxel, and Rapamycin in the high-risk group were much lower than those in the low-risk group, suggesting that patients with ccRCC in the high-risk group might benefit from the treatment of these drugs, while the IC50 levels of Lapatinib in high-risk group were much higher than that in the low-risk group, suggesting that patients with ccRCC in the low-risk group might benefit from the treatment of Lapatinib. Nevertheless, there were no obvious differences in the sensitivity of Axitinib and Pazopanib among two groups.

### Consensus clustering

**Figure 11:**
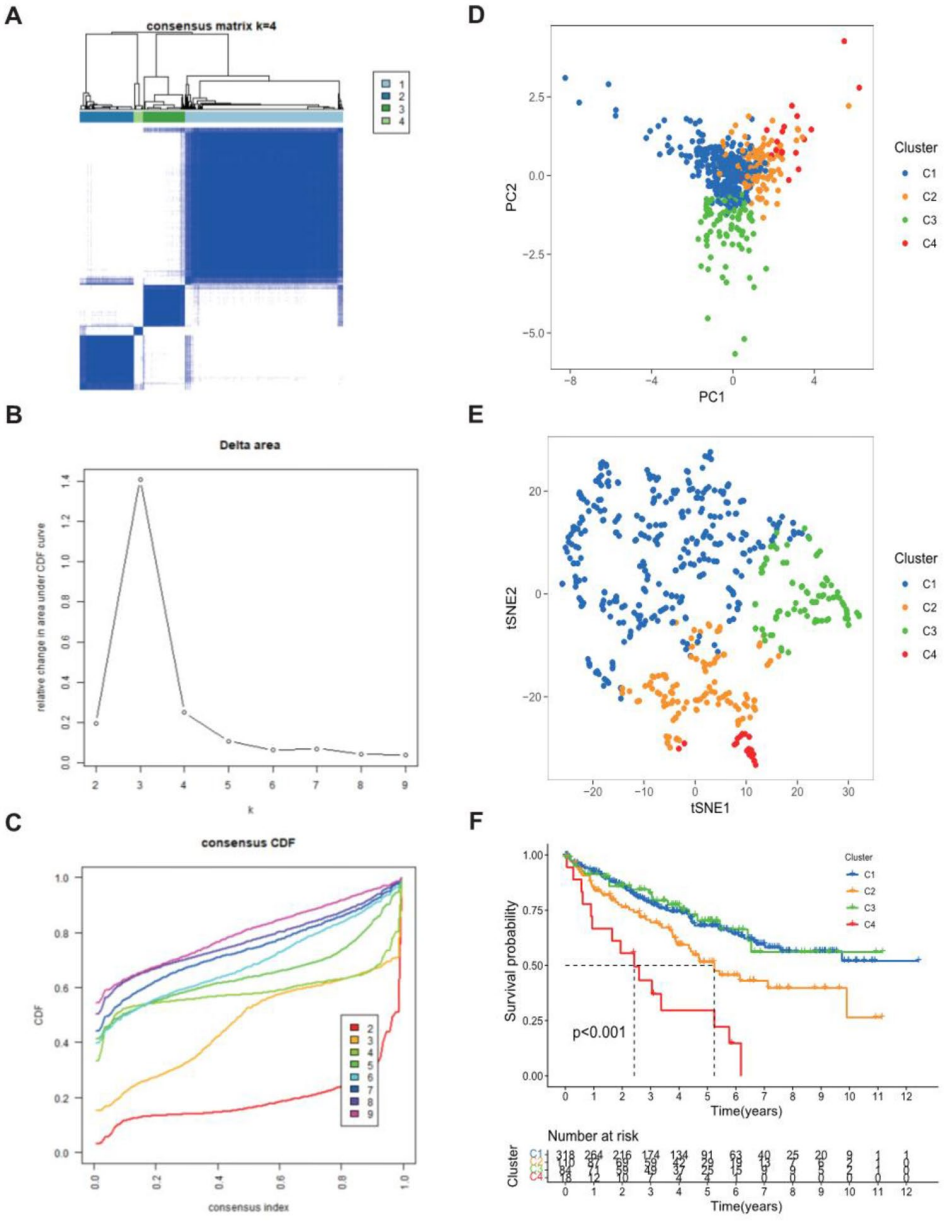
Consensus clustering for Notch-related lncRNA signature. (**A**) Consensus clustering matrix for k = 4; (**B**) Consensus clustering cumulative distribution function (CDF); (**C**) The relative change in area under CDF curve for k = 2–9; (**D**) PCA analysis among four clusters; (**E**) t-SNE analysis among four clusters; (**F**) Kaplan–Meier curve for evaluating the prognosis of four clusters.

**Figure 12:**
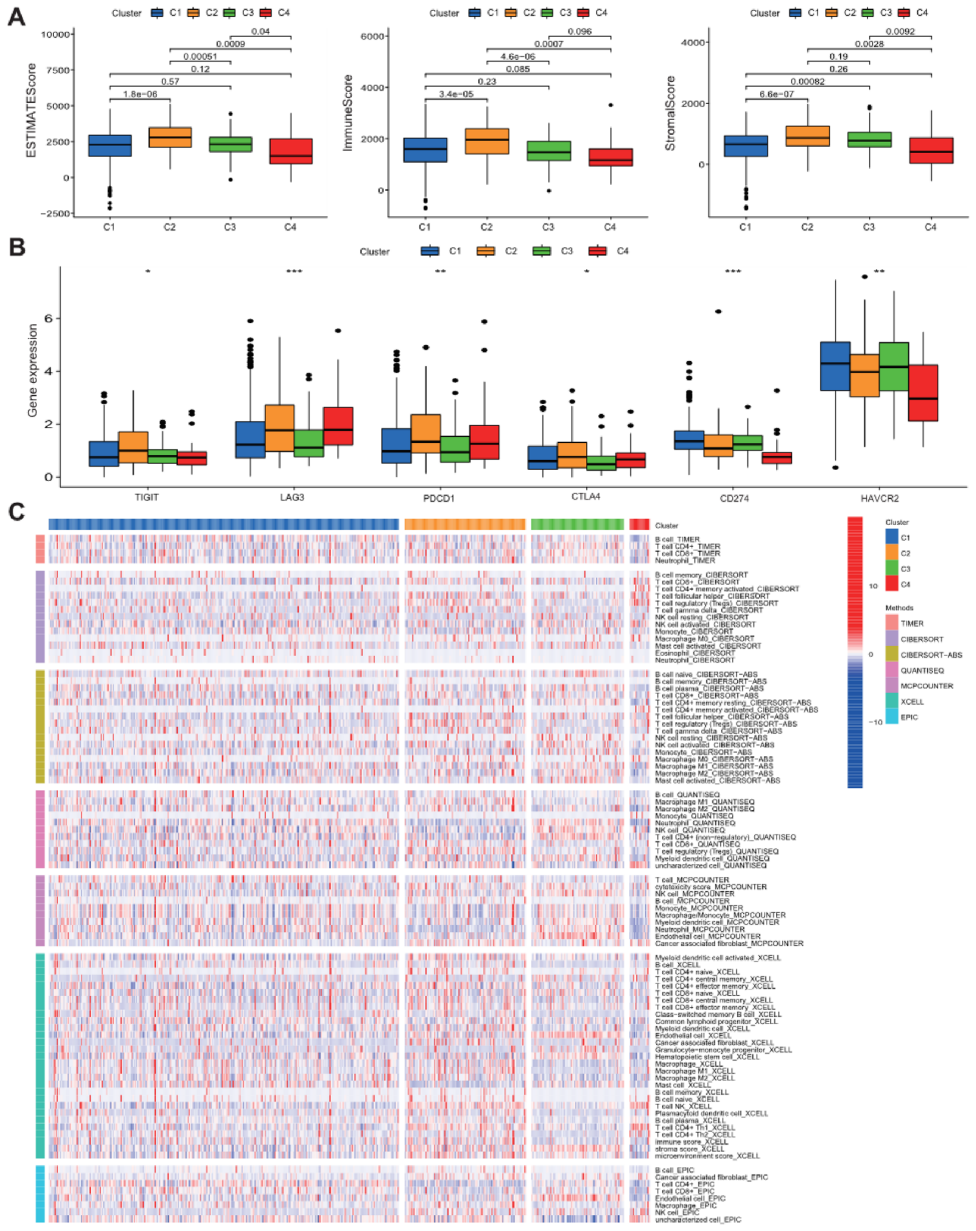
The immune characteristics of the prognostic lncRNA patterns between four clusters. (**A**) The expression of immune score, stromal cell score, and ESTIMATE Score among four clusters; (**B**) The expression of key immune checkpoints among four clusters; (**C**) The distribution of 22 immune cells infiltration among four clusters.

To develop a classification of ccRCC based on the expression level of risk score, unsupervised consensus clustering was performed on ccRCC patients in the total cohort. We found that k = 4 had the optimum clustering power, and patients with ccRCC were separated into four subgroups: Cluster 1 (n = 318), Cluster 2 (n = 110), Cluster 3 (n = 84), and Cluster 4 (n = 18) (Fig. 11A–C). PCA and t-SNE analyses also displayed the stability of clustering power between four subgroups (Fig. 11D,E). The results of Kaplan–Meier analysis showed that the overall survival of Cluster 1 and Cluster 3 were the best, followed by the Cluster 2, and the Cluster 4 being the worst (Fig. 11F). In addition, CD4 + T cells, T cell follicular helper, Tregs, macrophage M1 and M2, and neutrophil were the obvious immune infiltrating cells between these four clusters (Fig. 12C). The expression levels of LAG3, PD-1, and CTLA4 in cluster 2 and cluster 4 were much higher compared than those in cluster 1 and cluster 3 (Fig. 12B). The expression levels of HAVCR2 and PD-L1 in cluster 1 and cluster 3 were markedly higher than that in cluster 2, and cluster 4 being the lowest. The immune score in cluster 2 was the highest, the cluster 1, cluster3, and cluster4 being reduced in turn (Fig. 12A). The ESTIMATE score and stromal score in cluster 2 were the highest, followed by the cluster 3 and cluster 1, and cluster 4 being the lowest. All these results indicated the noticeable heterogeneity of ccRCC patients defined by the risk score.

## Discussion

Renal cell carcinoma (RCC) is essentially a metabolic disease characterized by a reprogramming of energetic metabolism^31–34^. In particular the metabolic flux through glycolysis is partitioned^35–37^, and mitochondrial bioenergetics and OxPhox are impaired, as well as lipid metabolism^35,38,39^. In this scenario it has been shown that Notch signaling (including some lncRNAs such asAGAP2-AS1) has a role in regulating cell metabolism^40,41^. In addition, renal cell carcinoma is one of the most immune-infiltrated tumors^42,43^. Emerging evidence suggests that the activation of specific metabolic pathway have a role in regulating angiogenesis and inflammatory signatures^44,45^. Features of the tumor microenvironment heavily affect disease biology and may affect responses to systemic therapy^46–49^. Notch signaling can modulate immune cell infiltration and regulate immunoflogosis^50^. To the best of my knowledge, lncRNA has been claimed to function as an important role in tumorigenesis, tumor progression, metastasis, and drug resistance. Recently, accumulating studies aimed to explore the value of lncRNA functioning as noninvasive biological markers for diagnosis, recurrence, prognosis, and forecasting the therapeutic effects. Furthermore, the lncRNA-stratified prognostic signature has been extensively used for predicting the prognosis and therapeutic response of patients with cancer, including ccRCC. Sun et al. constructed and verified a novel signature stratified by five immune-related lncRNA^51^, exhibiting the excellent capacity in survival prediction of patients with ccRCC. Dong et al. established a nine redox-related lncRNA signature correlated with overall survival in ccRCC. Additionally, Yu et al. identified novel lncRNA-based immune index for predicting the response to immune checkpoint inhibitor immunotherapy^52^.

In the present study, a total of 41 NSRlncRNAs were found to might be significantly correlated with tumorigenesis by integrated bioinformatic analyses including WGCNA, of which 13 NSRlncRNAs were identified to be associated with prognosis by univariate Cox analysis. Subsequently, LASSO regression analysis was conducted to establish a Notch-related lncRNA signature comprising of five lncRNAs (AC092611.2, NNT-AS1, AGAP2-AS1, AC147651.3, and AC007406.3), which displayed the favorable predictive value for prognosis in the training cohort, testing cohort, and entre cohort. ROC analyses also confirmed the feasibility and accuracy of our lncRNA-based signature. Furthermore, multivariate Cox analysis indicated that the lncRNA-based signature was an independent prognostic indicator. The risk score and other clinical variables (age, AJCC stage, and grade) were incorporated to construct a nomogram for predicting the outcomes and guiding decision-making.

In the current signature consisting of five NSRlncRNAs (AC092611.2, NNT-AS1, AGAP2-AS1, AC147651.3, and AC007406.3), several NSRlncRNAs have been claimed to be involved in the carcinogenesis including ccRCC, or have an influence on the prognosis of patients with malignant renal tumor. Among the five NSRlncRNAs, AC092611.2 and AC147651.3 were identified as autophagy- and immune-related lncRNAs in ccRCC by bioinformatic analysis, respectively^53,54^. Nevertheless, the biological function of AC007406.3 in tumor has never been explored. Lnc RNA NNT-AS1 functioned as a cancer promotor in various tumors. For example, NNT-AS1 has been shown to function as the oncogene role in cancer, which was elevated in non‐small cell lung cancer (NSCLC) and tightly correlated with the tumorigenic phenotypes and unfavorable prognosis^55^. NNT-AS1 was obviously upregulated in ccRCC samples and NNT-AS1 advanced the proliferation, invasion, and metastasis of ccRCC cells via sponging miR-137 to especially elevate the expression of YBX-1^56^. A meta-analysis suggested that the overexpression of NNT-AS1 was correlated with poor prognosis and advanced clinicopathologic characteristics in tumors^57^. AGAP2-AS1, a known cancer-related lncRNA activated by SP1 and RREB1, has been broadly upregulated in various types of tumors and correlated with the biological behaviors of cancer cells, including lung cancer^58,59^, breast cancer, gastric cancer^60^, pancreatic cancer^61^, hepatocellular cancer, esophageal cancer^62^, and ccRCC^63^.

The crucial role of immune infiltration in the tumorigenesis, progression, prognosis, and drug resistance of ccRCC has been broadly acknowledged. Infiltrated CD4 + T cells stimulated the proliferation of RCC cells via regulating TGFβ1/YBX1/HIF2α pathway^64^. The cytotoxic CD8 + T cells could attenuate the growth of cancer cells by targeting antigenic cancer cells^65^. Elevated peritumoral Tregs was associated with inferior outcomes in ccRCC^66^. A meta-analysis showed that the higher infiltration of FoxP3 + Tregs was notably correlated with shorter prognosis in several solid tumors^67^. We first explored the relationship between the lncRNA-based prognostic signature and tumor microenvironment. Our results suggested that lncRNA-based signature displayed the positive correlation with stromal score, immune score, and ESTIMATE score. Furthermore, we also found the obvious differences of the abundances of various immune cell between high-risk group and low-risk group, which implied that the Notch-related lncRNA signature was correlated with immune infiltration. Targeted therapy and immunotherapy were crucial for the treatments of advanced or metastatic ccRCC owing to the unsensitive to radiotherapy and chemotherapy. Hence, we further investigated the response to immunotherapy and targeted drugs among two groups. The results suggested that ccRCC patients in high-risk group were much more sensitive to targeted drugs, including Sunitinib, Sorafenib, Gefitinib, Erlotinib, Pazopanib, Lapatinib, Rapamycin, and Temsirolimus. Moreover, immune checkpoint inhibitors immunotherapy, an emerging antitumor therapy, has been authorized for various malignancies. In our study, the expressions of PD-L1, HAVCR2, PD-1, LAG-3, TIGIT, and CTLA-4 were much more markedly elevated in the high-risk group than those in the low-risk group, suggesting that patients in the high-risk group might be suitable for immunotherapy. All these results uncovered that our constructed prognostic signature might optimize clinical therapeutic options and serve as potential biomarker.

Of course, several disadvantages to our study also should be acknowledged. First, the development and verification of the prognostic model were mainly depended on only one retrospective cohort from TCGA dataset, and much more prospective data for verification should be essential. Additionally, all the results of our research were acquired via bioinformatic methods, which should be further experimental validation.

In conclusion, we identified a robust prognostic signature containing five Notch signaling-related lncRNAs. Moreover, this prognostic signature was of great clinical power for evaluating tumor immune microenvironment and providing individualized therapeutic strategies in patients with ccRCC.

### Supplementary Information

Supplementary Figures.

## Data Availability

The corresponding data in the present study were downloaded from TCGA (https:// por-tal.gdc.cancer.gov/),MSigDB (https://www.gsea-msigdb.org/gsea/msigdb), and GEO (https://www.ncbi.nlm.nih.gov/geo/).

## Abbreviations

CcRCC: Clear cell renal cell carcinoma
TCGA: The Cancer Genome Atlas
GEO: Gene Expression Omnibus
LncRNA: Long non-coding RNA
NSRlncRNA: Notch signaling-related lncRNA
WGCNA: Weighted gene co-expression network analysis
FDR: False discovery rate
FC: Fold change
GO: Gene ontology
