# Supplementary material for: A Notch signaling-related lncRNA signature for predicting prognosis and therapeutic response in clear cell renal cell carcinoma
Source: Sci Rep. 2023 Nov 30;13:21141. doi: 10.1038/s41598-023-48596-2 (PMC10689792; doi:10.1038/s41598-023-48596-2)

Supplementary Figure 1

The correlation between the risk scores and clinical variables. (A)Heatmap showed the relative expression of the risk score in ccRCC patients in various clinical various, including age, sex, tumor grade, and AJCC stage. ****P*＜0.001＜***P*＜0.01＜**P*＜0.05; (B) The relative expression of the risk score in subgroups stratified by age, sex, tumor grade, and AJCC stage.


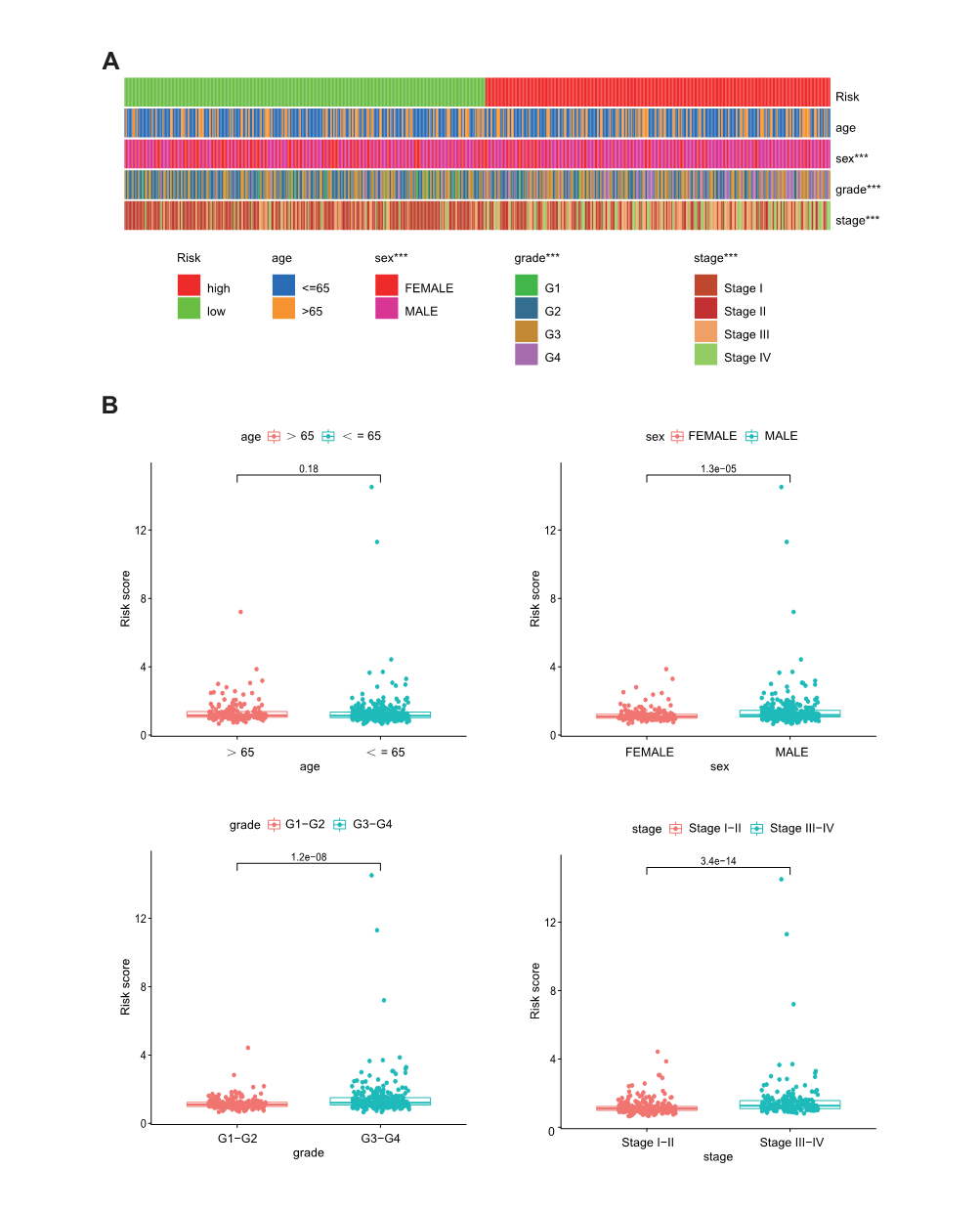


Supplementary Figure 2

The relative expression of key immune checkpoints between high- and low-risk groups in the total TCGA dataset.


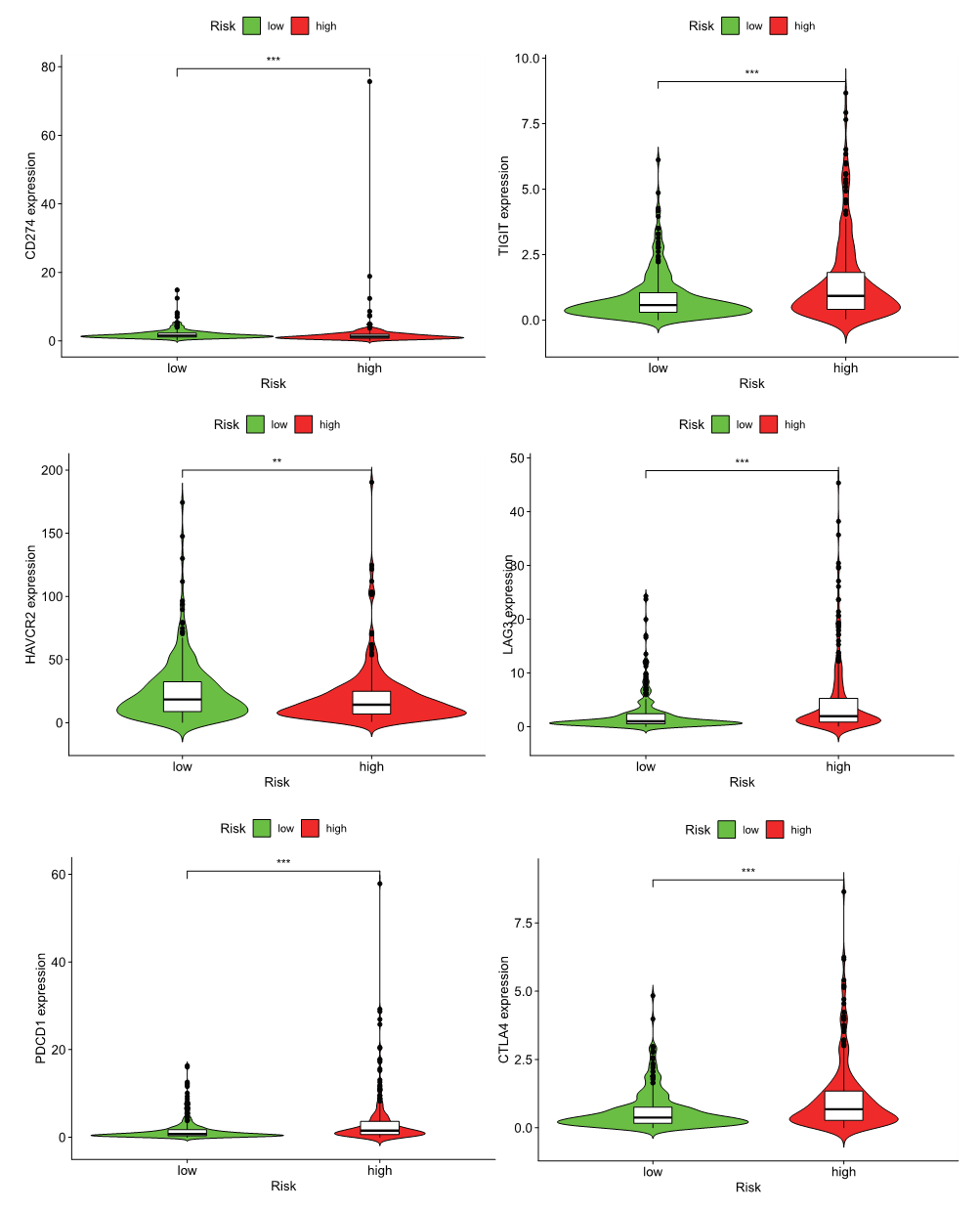


Supplementary Figure 3

Functional enrichment analyses. (A) The potential pathways by Gene Set Enrichment Analysis (GSEA) between the high- and low-risk groups; (B) GO analysis of the differentially expressed genes between the high- and low-risk groups; (C) KEGG analysis of the differentially expressed genes between the high- and low-risk groups.


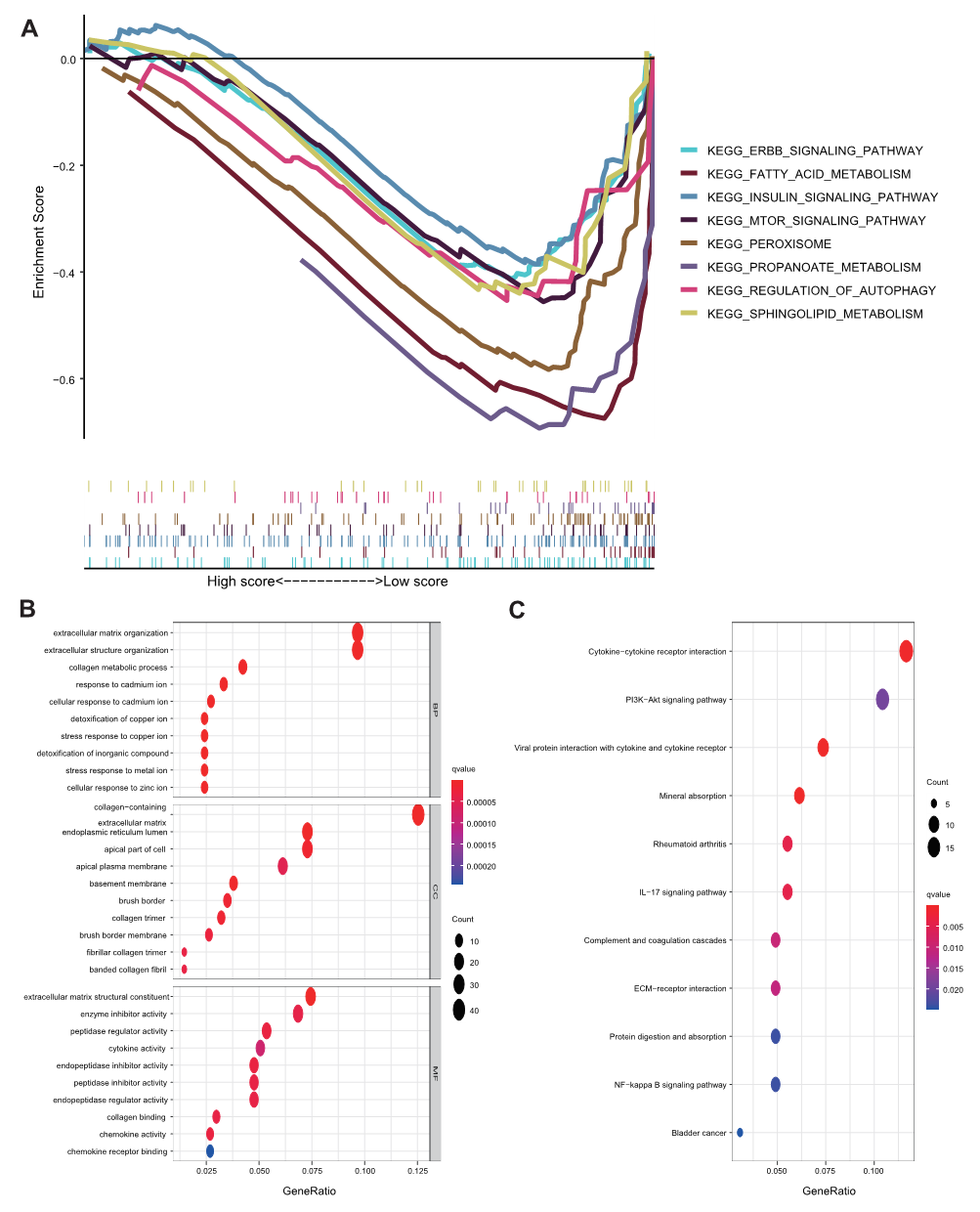

Supplement: Supplementary file 1 — Supplementary Figures. [file 41598_2023_48596_MOESM1_ESM.docx]
